# Supplementary material for: Novel community health worker strategy for HIV service engagement in a hyperendemic community in Rakai, Uganda: A pragmatic, cluster-randomized trial
Source: PLoS Med. 2021 Jan 6;18(1):e1003475. doi: 10.1371/journal.pmed.1003475 (PMC7787382; doi:10.1371/journal.pmed.1003475)
Supplement: S1 Text — (DOCX) [file pmed.1003475.s002.docx]

**Supplementary Appendix**

**Figure A. Health Scouts conceptual framework.**

**Figure B. Example screenshots from Health Scout smartphone application.**


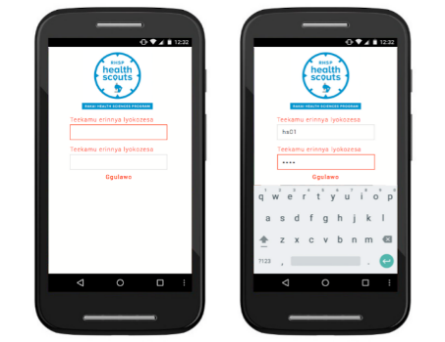

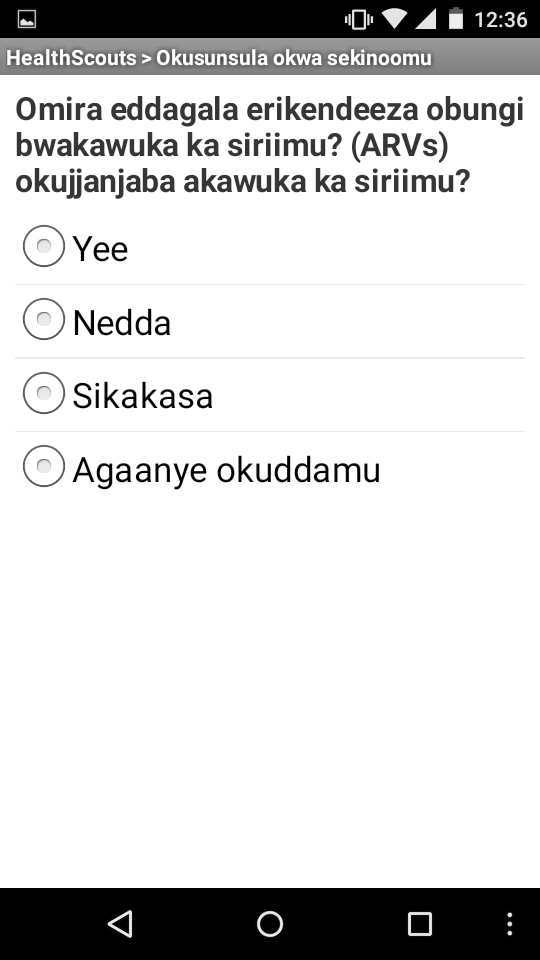


**Figure C. Example of phone application counseling module flow and content: HIV-positive, not in care.** Each rectangle represents one screenshot of messages/prompts. Health Scouts were to check off each item after engaging on that topic with the client. All items on a screen had to be checked prior to the Health Scout moving on to the next screen.

**Table A. Detailed algorithm for the mLAKE (mHealth Lakefolk Actively Keeping Engaged) application, including counseling messages.** Note: The PrEP module was added in 2017 when PrEP became available in this community.

| **#** | **Item** | | **Coding** | | **Response** | | | | | | **Variable Name** |  |
| --- | --- | --- | --- | --- | --- | --- | --- | --- | --- | --- | --- | --- |
| **Initial Household Screen**-to discern how many are eligible for counseling | | | | | | | | | | | |  |
| 1.1 | Record counseling attempt location. | [Button (GPS)] | | 🡪🡪🡪🡪🡪🡪🡪🡪🡪🡪 | | | | | GPS | | |  |
| 1.2 | O New location?  O Follow-up location? | [Checkbox] | | 🡪🡪🡪🡪🡪🡪🡪🡪🡪🡪 | | | | | NEW | | |  |
| 1.3 | Anyone present? | Yes…………………. 1  No………………….. 0 | | If 1, skip to 1.5  If 0, proceed to 1.4 | | | | | PRESENT | | |  |
| 1.4 | This attempt to locate is complete. | [Button (Save form and close application.)] | | If button pressed, save form and close application. | | | | | END1 | | |  |
| 1.5 | Greet person “My name is ___ and I am a xxxx”. | [Checkbox] | | 🡪🡪🡪🡪🡪🡪🡪🡪🡪🡪 | | | | | GREET | | |  |
| 1.6 | Confirm that there are persons age 15 or older present for possible counseling. | Yes…………………. 1  No/Not sure………….. 0 | | If 1, skip to 1.8  If 0, proceed to 1.7 | | | | | FIFTEEN | | |  |
| 1.7 | Thank person(s) for their time and let them know your services are only for those age 15 or older. | [Button (Save form and close application.)] | | If button pressed, save form and close application. | | | | | END2 | | |  |
| **Oral Consent/Assent Process with resident(s), one at a time, in private. If obtained proceed with Individual Screening.** | | | | | | | | | | | |  |
| **Individual Screening-**performed client by client, in private | | | | | | | | | | | |  |
| 2.0 | O Let the client know that you need to start by asking some important questions.  O All of the information they share will be kept private.  O There are no wrong or right answers. You are just trying to find out how best to help them. | | [Checkbox] | | | | 🡪🡪🡪🡪🡪🡪🡪🡪🡪🡪 | | | | IS1 |  |
| 2.1 | Have you ever participated in this counseling service before? | | Yes…………………. 1  No………………….. 0  Not sure……………. 77 | | | | If 1, proceed to 2.3  If 0 or 77, 🡪🡪🡪🡪🡪🡪🡪 | | | | NEW2 |  |
| 2.2 | Begin a new Log Book sheet for this participant. The Participant ID is [Display Participant ID] | | [Checkbox] | | | | Proceed to 2.4 | | | | NEWLOG |  |
| 2.3 | Locate the Log Book sheet for this participant. The Participant ID is [Display Participant ID] | | [Checkbox] | | | | 🡪🡪🡪🡪🡪🡪🡪🡪🡪🡪 | | | | OLDLOG |  |
| 2.4 | Age | | [Number] | | | | 🡪🡪🡪🡪🡪🡪🡪🡪🡪🡪 | | | | AGE |  |
| 2.5 | Gender | | Male…………………. 1  Female………………… 2 | | | | If 1, suppress question 2.15  If 2, suppress question 2.14 | | | | SEX |  |
| 2.6 | Marital Status | | Single………………. 1  Divorced…………… 2  Widowed…………... 3  Married (Non-polygamous)….…… 4  Married (Polygamous).. 5  Other……………….. 6 | | | | 🡪🡪🡪🡪🡪🡪🡪🡪🡪🡪 | | | | MARITAL |  |
| 2.7 | Occupations (may select more than one). | | Fishing on the Lake…. 1  Fish factory worker….. 2  Agriculture………. 3 Housework in your  own home………….. 4  Housekeeper (for  relative or employer) … 5 Home brewing……… 6  Government/clerical.. 7  Teacher……. 8  Student….. 9 Military/police…… 10 Shopkeeper….. 11 Trading/vending….. 12 Bar worker or owner…. 13 Trucker/Transport…. 14  Other……. 15 | | | | 🡪🡪🡪🡪🡪🡪🡪🡪🡪🡪 | | | | OCCUP |  |
| 2.8 | Have you ever been tested for HIV? | | Yes…………………. 1  No………………….. 0  Not sure……………. 77  Declined to answer.. 88 | | | | If 0, then skip to 2.14 OR 2.15  Else, 🡪🡪🡪🡪🡪🡪 | | | | TESTED |  |
| 2.9 | Have you been HIV tested in the past 12 months? | | Yes…………………. 1  No………………….. 0  Not sure……………. 77  Declined to answer.. 88 | | | | 🡪🡪🡪🡪🡪🡪🡪🡪🡪🡪 | | | | TESTED12 |  |
| 2.10 | Have you ever tested positive for HIV? | | Yes…………………. 1  No………………….. 0  Not sure……………. 77  Declined to answer.. 88 | | | | If 0, then skip to 2.14 OR 2.15  Else, 🡪🡪🡪🡪🡪🡪  If 1, then skip 2.19 | | | | HIVPOS |  |
| 2.11 | Are you taking Septrin? | | Yes…………………. 1  No………………….. 0  Not sure……………. 77  Declined to answer.. 88 | | | | 🡪🡪🡪🡪🡪🡪🡪🡪🡪🡪 | | | | TAKESEP |  |
| 2.12 | Are you taking antiretrovirals for HIV? | | Yes…………………. 1  No………………….. 0  Not sure……………. 77  Declined to answer.. 88 | | | | 🡪🡪🡪🡪🡪🡪🡪🡪🡪🡪 | | | | TAKEART |  |
| 2.13 | When did you last go to a clinic or hospital for HIV care? | | Within the past 6  months…………. 1  Between 6-12 months.. 2  Over 12 months ago.. 3  Never gone…... 4  Not sure….. 77  Declined to answer.. 88 | | | | 🡪🡪🡪🡪🡪🡪🡪🡪🡪🡪 | | | | CARE |  |
| 2.14 | Are you circumcised? | | Yes…………………. 1  No………………….. 0  Not sure……………. 77  Declined to answer.. 88 | | | | Skip to 2.16 | | | | CIRC |  |
| 2.15 | Are you pregnant? | | Yes…………………. 1  No………………….. 0  Not sure……………. 77 Declined to answer.. 88 | | | | 🡪🡪🡪🡪🡪🡪🡪🡪🡪🡪 | | | | PREG |  |
| 2.16 | Have you had sex without a condom in the past 12 months? | | Yes…………………. 1  No………………….. 0  Not sure……………. 77  Declined to answer.. 88 | | | | 🡪🡪🡪🡪🡪🡪🡪🡪🡪🡪 | | | | CONDOM |  |
| 2.17 | Over the last year (12 months), how much time do you spend staying outside of X? | | Most of the year….. 1  About half of the year.. 2  A few months……. 3  A few weeks……… 4  A few days…….. 5  Never gone……… 6  Declined to answer.. 88 | | | | 🡪🡪🡪🡪🡪🡪🡪🡪🡪🡪 | | | | MOBILITY |  |
| 2.18 | Do you have any children? | | Yes…………………. 1  No………………….. 0  Declined to answer.. 88 | | | | 🡪🡪🡪🡪🡪🡪🡪🡪🡪🡪 | | | | CHILD |  |
| 2.19 | Are you taking Pre-Exposure Prophylaxis, also known as PrEP? | | Yes…………………. 1  No………………….. 0  Not sure……………. 77  Declined to answer.. 88 | | | | 🡪🡪🡪🡪🡪🡪🡪🡪🡪🡪 | | | | PREP |  |
|  | **Module Activation Algorithms**-based on responses above, the relevant HIV risk modules are activated below. | | | | | | | | | | |  |
| A | IF [[2.8=(0 OR 77 OR 88) OR 2.9=(0 OR 77 OR 88)] AND [2.5=1 OR 2.15=(0)]] THEN activate Module A | | | | | | | | | | |  |
|  | IF [[“Have you ever been tested for HIV” is No (0) or Not Sure (77) or Declined to Answer (88) OR “Have you been HIV tested in the last 12 months” is No (0) or Not Sure (77) or Declined to Answer (88)] AND “Have you ever tested positive for HIV” is No (0) or Not Sure (77) or Declined to Answer (88) AND “Are you pregnant” is No (0)] THEN activate Module A (HIV serostatus unknown or no recent HIV test, Male or Female not pregnant) | | | | | | | | | | |  |
| B | IF [2.8=[(0 OR 77 OR 88) OR 2.9=(0 OR 77 OR 88)] AND 2.15=(1 OR 77 OR 88)] THEN activate Module B | | | | | | | | | | |  |
|  | IF [“Have you ever been tested for HIV” is No (0) or Not Sure (77) or Declined to Answer (88) OR “Have you been HIV tested in the last 12 months” is No (0) or Not Sure (77) or Declined to Answer (88) AND “Have you ever tested positive for HIV” is No (0) or Not Sure (77) or Declined to Answer (88) AND “Are you pregnant” is Yes (1) or Not Sure (77) or Declined to Answer (88)] THEN activated Module B (HIV serostatus unknown or no recent HIV test, Female pregnant) | | | | | | | | | | |  |
| C | IF [2.5=1 AND 2.14=(0 OR 77 OR 88)] THEN activate Module C | | | | | | | | | | |  |
|  | IF [Gender is Male (1) AND “Are you circumcised” is No (0) or Not Sure (77) or Declined to Answer (88)] THEN activate Module C (Male, MMC-) | | | | | | | | | | |  |
| D | IF [[2.10=1 AND [2.11=(0 OR 77 OR 88) OR 2.12=(0 OR 77 OR 88)] AND 2.13=(2 OR 3 OR 4 OR 77 OR 88)]] THEN activate Module D | | | | | | | | | | |  |
|  | IF [“Have you ever tested positive for HIV” is Yes (1) AND “Are you taking Septrin” is No (0) or Not Sure (77) or Declined to Answer (88) OR “Are you taking antiretrovirals for HIV” is No (0) or Not Sure (77) or Declined to Answer (88) OR “When did you last go to a clinic or hospital for HIV care” is Between 6 and 12 Months Ago (2) or Over 12 Months Ago (3) or Never Gone (4) or Not Sure (77) or Declined to Answer (880] THEN activate Module D (HIV-positive, Not in care) | | | | | | | | | | |  |
| E | IF [2.10=1 AND 2.12=(0 OR 77 OR 88) AND 2.13=1] THEN activate Module E | | | | | | | | | | |  |
|  | IF [“Have you ever tested positive for HIV” is Yes (1) AND “Are you taking antiretrovirals for HIV” is No (0) or Not Sure (77) or Declined to Answer (88) AND “When did you last go to a clinic or hospital for HIV care” is Within the past 6 months (1)] THEN activate Module E (HIV-positive, In care, Not on ART) | | | | | | | | | | |  |
| F | IF [2.10=1 AND 2.12=1] THEN activate Module F | | | | | | | | | | |  |
|  | IF [“Have you ever tested positive for HIV” is Yes (1) AND “Are you taking antiretrovirals for HIV” is Yes (1)] THEN activate Module F (HIV-positive, On ART) | | | | | | | | | | |  |
| G | IF [2.15=(1 OR 77 OR 88) AND 2.10=1] THEN activate Module G | | | | | | | | | | |  |
|  | IF [“Are You Pregnant” is Yes (1) Not Sure (77) or Declined to Answer (88) AND “Have you ever tested positive for HIV” is Yes (1)] THEN Activate Module G (Pregnant +, HIV +) | | | | | | | | | | |  |
| H | IF [2.5=1 AND 2.16=(1 OR 77 OR 88)] THEN activate Module H | | | | | | | | | | |  |
|  | IF [Gender is Male (1) AND “Have you had sex without a condom in the last 12 months” is Yes (1) or Not Sure (77) or Declined to Answer (88)] THEN activate module H(Risky Sex+, Male+) | | | | | | | | | | |  |
| I | IF [2.5=2 AND 2.16=(1 OR 77 OR 88)] THEN activate Module I | | | | | | | | | | |  |
|  | IF {Gender is Female (2) AND “Have you had sex without a condom in the last 12 months” is Yes (1) or Not Sure (77) or Declined to Answer (88)] THEN activate Module I (Risky Sex+, Female+) | | | | | | | | | | |  |
| JK | ALWAYS activate Modules J and K | | | | | | | | | | |  |
| L | IF [Module A or B Activated] OR [2.10=0 OR 77 OR 88] AND 2[2.19=0 OR 77 OR 88] THEN Activate Module L | | | | | | | | | | |  |
|  | IF [Module A or B Activated] OR [“Have you ever tested positive for HIV” is No (0) or Not Sure (77) or Declined to Answer (88)] AND [“Are you taking Pre-Exposure Prophylaxis, also known as PrEP?” is No (0) or Not Sure (77) or Declined to Answer (88)] THEN Activate Module L | | | | | | | | | | |  |
| M | IF 2.19=1 THEN Activate Module M | | | | | | | | | | |  |
|  | IF [“Are you taking Pre-Exposure Prophylaxis, also known as PrEP?” is Yes (1)] THEN Activate Module M | | | | | | | | | | |  |
|  | **MODULES**-modules are activated based on responses to individual screening questions above. **Note: I, M, B in parentheses indicates relevant domains of the sIMB module.** | | | | | | | | | | |  |
| **A** | **HIV serostatus unknown or no recent HIV test, Male or Female not pregnant** | |  | | |  | | | |  | |  |
| 3.1 | Please ask the client the following questions using the motivational interviewing skills you have learned. | |  | | | 🡪🡪🡪🡪🡪🡪🡪🡪🡪🡪 | | | |  | |  |
| 3.2 | **Information**  O *Can you tell me what you know about HIV testing?* | | [Checkbox] | | | 🡪🡪🡪🡪🡪🡪🡪🡪🡪🡪 | | | | **AINFO1** | |  |
| 3.3 | **Motivation**  *O What would be some benefits to getting an HIV test?*  *O What are your concerns about getting an HIV test?*  *O What have been the not so good things that have happened or may happen by not getting tested?* | | [Checkbox] | | | 🡪🡪🡪🡪🡪🡪🡪🡪🡪🡪 | | | | **AMOTI1** | |  |
| 3.4 | **Behavioral Skills**  O*How confident are you that you could get an HIV test if you wanted to?*  *O What would make getting the test easy for you to do if you wanted to?*  *O What would make getting the test difficult for you to do if you wanted to?* | | [Checkbox] | | | 🡪🡪🡪🡪🡪🡪🡪🡪🡪🡪 | | | | **ABEHA1** | |  |
| 3.5 | **Intentions**  *O Given how you feel right now, would you want to get HIV tested within the next month?* | | Yes…………………. 1  No………………….. 0  Not sure……………. 77 | | | If 1, then skip to 3.7  Else, 🡪🡪🡪🡪🡪🡪 | | | | **AMOTI2** | |  |
| 3.6 | **Targets**  *O What would need to change for you to want to get tested?* | | [Checkbox] | | | 🡪🡪🡪🡪🡪🡪🡪🡪🡪🡪 | | | | **AMOTI3** | |  |
| 3.7 | *Some people are ready to get tested some are not ready right now. Most people think that it is helpful to have information, no matter how ready they are.*  *So if it is okay with you, I will share some additional information with you:*  O HIV Testing is free. **[I]** O The RHSP X clinic is the nearest HIV testing location.**[I]** O Support is available, myself and others can support and help you. **[M]** O Facilitated disclosure is available if needed. **[B]** | | [Checkbox] | | | 🡪🡪🡪🡪🡪🡪🡪🡪🡪🡪 | | | | **A1** | |  |
| **B** | **HIV serostatus unknown or no recent HIV test, Female pregnant** | |  | | |  | | | |  | |  |
| 4.1 | Please ask the client the following questions using the motivational interviewing skills you have learned. | |  | | | 🡪🡪🡪🡪🡪🡪🡪🡪🡪🡪 | | | |  | |  |
| 4.2 | **Information**  O *Can you tell me what you know about HIV testing?*  O *Can you tell me what you know about HIV testing when a woman is pregnant?* | | [Checkbox] | | | 🡪🡪🡪🡪🡪🡪🡪🡪🡪🡪 | | | | **BINFO1** | |  |
| 4.3 | **Motivation**  *O What would be some benefits to getting an HIV test?*  *O What would be some benefits to getting an HIV test when pregnant?*  *O What are your concerns about getting an HIV test?*  *O What have been the not so good things that have happened or may happen by not getting tested?* | | [Checkbox] | | | 🡪🡪🡪🡪🡪🡪🡪🡪🡪🡪 | | | | **BMOTI1** | |  |
| 4.4 | **Behavioral Skills**  O*How confident are you that you could get an HIV test if you wanted to?*  *O What would make getting the test easy for you to do if you wanted to?*  *O What would make getting the test difficult for you to do if you wanted to?* | | [Checkbox] | | | 🡪🡪🡪🡪🡪🡪🡪🡪🡪🡪 | | | | **BBEHA1** | |  |
| 4.5 | **Intentions**  *O Given how you feel right now, would you want to get HIV tested within the next month?* | | Yes…………………. 1  No………………….. 0  Not sure……………. 77 | | | If 1, then skip to 4.7  Else, 🡪🡪🡪🡪🡪🡪 | | | | **BMOTI2** | |  |
| 4.6 | **Targets**  *O What would need to change for you to want to get tested?* | | [Checkbox] | | | 🡪🡪🡪🡪🡪🡪🡪🡪🡪🡪 | | | | **BMOTI3** | |  |
| 4.7 | *Some people are ready to get tested some are not ready right now. Most people think that it is helpful to have information, no matter how ready they are.*  *So if it is okay with you, I will share some additional information with you:*  O HIV Testing is free. **[I]** O The RHSP X clinic is the nearest HIV testing location.**[I]** O Support is available, myself and others can support and help you. **[M]** O Facilitated disclosure is available if needed. **[B]**  O For pregnant women, getting tested for HIV is a great step to making sure they have a healthy baby. **[I,M]** | | [Checkbox] | | | 🡪🡪🡪🡪🡪🡪🡪🡪🡪🡪 | | | | **B1** | |  |
| **C** | **Male, MMC-** | |  | | |  | | | |  | |  |
| 5.1 | Please ask the client the following questions using the motivational interviewing skills you have learned. | |  | | | 🡪🡪🡪🡪🡪🡪🡪🡪🡪🡪 | | | |  | |  |
| 5.2 | **Information**  O *Can you tell me what you know about MMC?* | | [Checkbox] | | | 🡪🡪🡪🡪🡪🡪🡪🡪🡪🡪 | | | | **CINFO1** | |  |
| 5.3 | **Motivation**  *O What would be some benefits of MMC?*  *O What are your concerns about circumcision?*  *O What have been the not so good things that have happened or may happen by not being circumcised?* | | [Checkbox] | | | 🡪🡪🡪🡪🡪🡪🡪🡪🡪🡪 | | | | **CMOTI1** | |  |
| 5.4 | **Behavioral Skills**  O *How confident are you that you could get MMC if you wanted to?*  *O What are things that would make it difficult to get MMC even if you wanted to?* | | [Checkbox] | | | 🡪🡪🡪🡪🡪🡪🡪🡪🡪🡪 | | | | **CBEHA1** | |  |
| 5.5 | **Intentions**  *O Given how you feel right now, would you want MMC within the next month?* | | Yes…………………. 1  No………………….. 0  Not sure……………. 77 | | | If 1, then skip to 5.7  Else, 🡪🡪🡪🡪🡪🡪 | | | | **CMOTI2** | |  |
| 5.6 | **Targets**  *O What would need to change for you to want to get MMC?* | | [Checkbox] | | | 🡪🡪🡪🡪🡪🡪🡪🡪🡪🡪 | | | | **CMOTI3** | |  |
| 5.7 | *Some people are ready to get circumcised and some are not ready right now. Most people think that it is helpful to have information, no matter how ready they are. So if it is okay with you, I will share some additional information with you:*  O If you are concerned about pain, we now have some new ways to minimize pain. **[I]** | | [Checkbox] | | | 🡪🡪🡪🡪🡪🡪🡪🡪🡪🡪 | | | | **C1** | |  |
| **D** | **HIV-positive, Not in care** | |  | | |  | | | |  | |  |
| 6.1 | Please ask the client the following questions using the motivational interviewing skills you have learned. | |  | | | 🡪🡪🡪🡪🡪🡪🡪🡪🡪🡪 | | | |  | |  |
| 6.2 | **Information**  O *Can you tell me what you know about HIV care?*  O*What do you know about the availability of HIV care when you are not staying in X?* | | [Checkbox] | | | 🡪🡪🡪🡪🡪🡪🡪🡪🡪🡪 | | | | **DINFO1** | |  |
| 6.3 | **Motivation**  *O What would be some benefits to getting into HIV care?*  *O What are your concerns about HIV care?*  *O What are your concerns about getting health care when you are not staying in X?*  *O What have been the not so good things that have happened or may happen by not getting into care?* | | [Checkbox] | | | 🡪🡪🡪🡪🡪🡪🡪🡪🡪🡪 | | | | **DMOTI1** | |  |
| 6.4 | **Behavioral Skills**  O*How confident are you that you could get HIV care if you wanted to?*  *O What are things that would make it difficult to get HIV care even if you wanted to?* | | [Checkbox] | | | 🡪🡪🡪🡪🡪🡪🡪🡪🡪🡪 | | | | **DBEHA1** | |  |
| 6.5 | **Intentions**  *O Given how you feel right now, would you want to get HIV care?*  *O Given how you feel right now, do you plan on getting HIV care when you travel?* | | Yes…………………. 1  No………………….. 0  Not sure……………. 77  Yes…………………. 1  No………………….. 0  Not sure……………. 77 | | | If 1 for both, then skip to 6.7  Else, 🡪🡪🡪🡪🡪🡪 | | | | **DMOTI2**  **DMOTI2B** | |  |
| 6.6 | **Targets**  *O What would need to change for you to want to get HIV care either in X or when traveling?* | | [Checkbox] | | | 🡪🡪🡪🡪🡪🡪🡪🡪🡪🡪 | | | | **DMOTI3** | |  |
| 6.7 | *Some people are ready to get in care and some are not ready right now. Most people think that it is helpful to have information, no matter how ready they are. So if it is okay with you, I will share some additional information with you:*  O HIV Care is free at the RHSP clinic. **[I]**  O If you need a referral to another health clinic while you are traveling, I can help you. **[I]**  O If you have children, having them tested for HIV is a great step to making sure they are healthy. **[I,M]** | | [Checkbox] | | | 🡪🡪🡪🡪🡪🡪🡪🡪🡪🡪 | | | | **D1** | |  |
| **E** | **HIV-positive, In care, Not on ART** | |  | | |  | | | |  | |  |
| 7.1 | Please ask the client the following questions using the motivational interviewing skills you have learned. | |  | | | 🡪🡪🡪🡪🡪🡪🡪🡪🡪🡪 | | | |  | |  |
| 7.2 | **Information**  O *Can you tell me what you know about ART?*  O *What do you know about the availability of ART when you are not staying in X?* | | [Checkbox] | | | 🡪🡪🡪🡪🡪🡪🡪🡪🡪🡪 | | | | **EINFO1** | |  |
| 7.3 | **Motivation**  *O What would be some benefits to getting ART?*  *O What are your concerns about ART?*  *O What have been the not so good things that have happened or may happen by not getting ART?* | | [Checkbox] | | | 🡪🡪🡪🡪🡪🡪🡪🡪🡪🡪 | | | | **EMOTI1** | |  |
| 7.4 | **Behavioral Skills**  O*How confident are you that you could get on ART if you wanted to?*  *O What are things that would make it difficult to get on ART even if you wanted to?* | | [Checkbox] | | | 🡪🡪🡪🡪🡪🡪🡪🡪🡪🡪 | | | | **EBEHA1** | |  |
| 7.5 | **Intentions**  *O Given how you feel right now, would you try to get started on ART?* | | Yes…………………. 1  No………………….. 0  Not sure……………. 77 | | | If 1, then skip to 7.7  Else, 🡪🡪🡪🡪🡪🡪 | | | | **EMOTI2** | |  |
| 7.6 | **Targets**  *O What would need to change for you to want to get started on ART?* | | [Checkbox] | | | 🡪🡪🡪🡪🡪🡪🡪🡪🡪🡪 | | | | **EMOTI3** | |  |
| 7.7 | *Some people are ready to start ART and some are not ready right now. Most people think that it is helpful to have information, no matter how ready they are. So if it is okay with you, I will share some additional information with you:*  O ART is free. **[I]**  O All persons with HIV in X are recommended to start ART. **[I]**  O To feel and look good, persons with HIV should take ART **[M]**  O If you have children, having them tested for HIV is a great step to making sure they are healthy. **[I,M]**  O If you need a referral to another health clinic while you are traveling, I can help you. **[I]** | | [Checkbox] | | | 🡪🡪🡪🡪🡪🡪🡪🡪🡪🡪 | | | | **E1** | |  |
| **F** | **HIV-positive, On ART** | |  | | |  | | | |  | |  |
| 8.1 | Please ask the client the following questions using the motivational interviewing skills you have learned. | |  | | | 🡪🡪🡪🡪🡪🡪🡪🡪🡪🡪 | | | |  | |  |
| 8.2 | **Information**  O *Can you tell me what you know about taking ART?*  O *What do you know about the availability of ART when you are not staying in X?* | | [Checkbox] | | | 🡪🡪🡪🡪🡪🡪🡪🡪🡪🡪 | | | | **FINFO1** | |  |
| 8.3 | **Motivation**  *O What would be some benefits to taking your ART meds?*  *O What are your concerns about your ART?*  *O What are your concerns about taking your ART when you are traveling?*  *O What have been the not so good things that have happened or may happen by not taking ART?* | | [Checkbox] | | | 🡪🡪🡪🡪🡪🡪🡪🡪🡪🡪 | | | | **FMOTI1** | |  |
| 8.4 | **Behavioral Skills**  O *If your goal was to take your ART every day what would help you achieve that goal?*  O *Who or what could help to make sure you stay on ARTeven while you travel?*  *O And what could get in the way of achieving these goals?* | | [Checkbox] | | | 🡪🡪🡪🡪🡪🡪🡪🡪🡪🡪 | | | | **FBEHA1** | |  |
| 8.5 | **Intentions**  *O Given how you feel right now, will you try to take your ART every day?*  *O Given how you feel right now, do you plan on staying on ART when you are not in X?* | | Yes…………………. 1  No………………….. 0  Not sure……………. 77  Yes…………………. 1  No………………….. 0  Not sure……………. 77 | | | If 1 to both, then skip to 8.7  Else, 🡪🡪🡪🡪🡪🡪 | | | | **FMOTI2**  **FMOTI2B** | |  |
| 8.6 | **Targets**  *O What would need to change for you to want to take your ART every day?*  *O What would need to change for you to want to stay on ART when you are not in X?* | | [Checkbox] | | | 🡪🡪🡪🡪🡪🡪🡪🡪🡪🡪 | | | | **FMOTI3** | |  |
| 8.7 | *If it is okay with you, I will share some additional information with you:*  O If you have children, having them tested for HIV is a great step to making sure they are healthy. **[I,M]**  O If you are on medicines, I can help you stay on them when you are traveling. **[B]**  O If you need a referral to another health clinic while you are traveling, I can help you. **[I]** | | [Checkbox] | | | 🡪🡪🡪🡪🡪🡪🡪🡪🡪🡪 | | | |  | |  |
| **G** | **Pregnant+, HIV positive** | | | | | | | | | | | |
| 9.1 | Please ask the client the following questions using the motivational interviewing skills you have learned. | |  | | | 🡪🡪🡪🡪🡪🡪🡪🡪🡪🡪 | | | |  | |  |
| 9.2 | **Information**  O *Can you tell me what you know about taking ART when you are pregnant?* | | [Checkbox] | | | 🡪🡪🡪🡪🡪🡪🡪🡪🡪🡪 | | | | **GINFO1** | |  |
| 9.3 | **Motivation**  *O What would be some benefits to taking ART when pregnant?*  *O What are your concerns about taking ART when pregnant?* | | [Checkbox] | | | 🡪🡪🡪🡪🡪🡪🡪🡪🡪🡪 | | | | **GMOTI1** | |  |
| 9.4 | **Behavioral Skills**  O *Who or what would support you in taking ART during your pregnancy?*  *O What could get in the way of this goal?* | | [Checkbox] | | | 🡪🡪🡪🡪🡪🡪🡪🡪🡪🡪 | | | | **GBEHA1** | |  |
| 9.5 | **Intentions**  *O Based on how you feel right now, do you plan to be on ART while pregnant?* | | Yes…………………. 1  No………………….. 0  Not sure……………. 77 | | | If 1, then skip to 9.7  Else, 🡪🡪🡪🡪🡪🡪 | | | | **GMOTI2** | |  |
| 9.6 | **Targets**  *O What would need to change for you to be on ART while pregnant?* | | [Checkbox] | | | 🡪🡪🡪🡪🡪🡪🡪🡪🡪🡪 | | | | **GMOTI3** | |  |
| 9.7 | *Some people are ready to get tested and some are not ready right now. Most people think that it is helpful to have information, no matter how ready they are. So if it is okay with you, I will share some additional information with you:*  O For pregnant women who are HIV-positive, getting and staying on ART is a great step to making sure they have a healthy baby. **[I,M]** | | [Checkbox] | | | 🡪🡪🡪🡪🡪🡪🡪🡪🡪🡪 | | | | **G1** | |  |
| **H** | **Risky Sex+, Male+** | |  | | |  | | | |  | |  |
| 10.1 | Please ask the client the following questions using the motivational interviewing skills you have learned. | |  | | | 🡪🡪🡪🡪🡪🡪🡪🡪🡪🡪 | | | |  | |  |
| 10.2 | **Information**  O *Can you tell me what you know about HIV transmission for men who do not always use a condom?* | | [Checkbox] | | | 🡪🡪🡪🡪🡪🡪🡪🡪🡪🡪 | | | | **HINFO1** | |  |
| 10.3 | **Motivation**  *O What would be some benefits to using a condom?*  *O What are your concerns about condoms?*  *O What have been the not so good things that have happened or may happen by not using condoms?* | | [Checkbox] | | | 🡪🡪🡪🡪🡪🡪🡪🡪🡪🡪 | | | | **HMOTI1** | |  |
| 10.4 | **Behavioral Skills**  O *If you decided to start using condoms, what would help you follow through on this goal?*  *O What could get in the way of using them?* | | [Checkbox] | | | 🡪🡪🡪🡪🡪🡪🡪🡪🡪🡪 | | | | **HBEHA1** | |  |
| 10.5 | **Intentions**  *O Given how you feel right now, do you plan on using condoms every time you have sex?* | | Yes…………………. 1  No………………….. 0  Not sure……………. 77 | | | If 1, then skip to 10.7  Else, 🡪🡪🡪🡪🡪🡪🡪 | | | | **HMOTI2** | |  |
| 10.6 | **Targets**  *O What would need to change for you to want to use condoms more?* | | [Checkbox] | | | 🡪🡪🡪🡪🡪🡪🡪🡪🡪🡪 | | | | **HMOTI3** | |  |
| 10.7 | *Some people are ready to use condoms and some are not ready right now. Most people think that it is helpful to have information, no matter how ready they are. So if it is okay with you, I will share some additional information with you:*  O Condoms are available for free through myself and the RHSP clinic. **[I, B]**  O Do you know how to use a condom? **[B]** | | [Checkbox] | | | 🡪🡪🡪🡪🡪🡪🡪🡪🡪🡪 | | | | **H1** | |  |
| **I** | **Risky Sex+, Female+** | |  | | |  | | | |  | |  |
| 11.1 | Please ask the client the following questions using the motivational interviewing skills you have learned. | |  | | | 🡪🡪🡪🡪🡪🡪🡪🡪🡪🡪 | | | |  | |  |
| 11.2 | **Information**  *O Can you tell me what you know about male condoms and HIV transmission?* | | [Checkbox] | | | 🡪🡪🡪🡪🡪🡪🡪🡪🡪🡪 | | | | **IINFO1** | |  |
| 11.3 | **Motivation**  *O What would be some benefits to you having your partner use a condom?*  *O What are your concerns about being able to use condoms with your partner or partners?*  *O What have been the not so good things that have happened or may happen by not having condoms used by your partner or partners?*  *O What are your concerns about discussing condom use with your partner?* | | [Checkbox] | | | 🡪🡪🡪🡪🡪🡪🡪🡪🡪🡪 | | | | **IMOTI1** | |  |
| 11.4 | **Behavioral Skills**  O*How confident are you that you could discuss condom use with your partner?*  *O What would make discussing condom use something difficult to do?* | | [Checkbox] | | | 🡪🡪🡪🡪🡪🡪🡪🡪🡪🡪 | | | | **IBEHA1** | |  |
| 11.5 | **Intentions**  *O Given how you feel right now, do you plan on discussing condom use with your partner?* | | Yes…………………. 1  No………………….. 0  Not sure……………. 77 | | | If 1, then skip to 11.7  Else, 🡪🡪🡪🡪🡪 | | | | **IMOTI2** | |  |
| 11.6 | **Targets**  *O What would need to change for you to want to use condoms more?* | | [Checkbox] | | | 🡪🡪🡪🡪🡪🡪🡪🡪🡪🡪 | | | | **IMOTI3** | |  |
| 11.7 | *Some people are ready to use condoms and some are not ready right now. Most people think that it is helpful to have information, no matter how ready they are. So if it is okay with you, I will share some additional information with you:*  O Condoms are available for free through myself and the RHSP clinic. **[I, B]**  O Do you know how to use a condom? **[B]** | | [Checkbox] | | | 🡪🡪🡪🡪🡪🡪🡪🡪🡪🡪 | | | | **I1** | |  |
| **L** | **PrEP Module for HIV serostatus unknown or no recent HIV test or HIV negative, Not taking PrEP** | |  | | |  | |  | | | |  |
| 14.1 | Please ask the client the following questions using the motivational interviewing skills you have learned. | |  | | | 🡪🡪🡪🡪🡪🡪🡪🡪🡪🡪 | |  | | | |  |
| 14.2 | **Information**  O *Can you tell me what you know about Pre-Exposure Prophylaxis, also known as PrEP?* | | [Checkbox] | | | 🡪🡪🡪🡪🡪🡪🡪🡪🡪🡪 | | **LINFO1** | | | |  |
| 14.3 | **Motivation**  *O What would be some benefits to taking PrEP?*  *O What are your concerns about PrEP?*  *O What have been the not so good things that have happened or may happen by not getting PrEP?* | | [Checkbox] | | | 🡪🡪🡪🡪🡪🡪🡪🡪🡪🡪 | | **LMOTI1** | | | |  |
| 14.4 | **Behavioral Skills**  O *How confident are you that you could get PrEP if you wanted to?*  *O What would make getting the PrEP easy for you if you wanted it?*  *O What would make getting PrEP difficult for you if you wanted it?* | | [Checkbox] | | | 🡪🡪🡪🡪🡪🡪🡪🡪🡪🡪 | | **LBEHA1** | | | |  |
| 14.5 | **Intentions**  *O Given how you feel right now, would you want PrEP within the next month?* | | Yes…………………. 1  No………………….. 0  Not sure……………. 77 | | | If 1, then skip to 3.7  Else, 🡪🡪🡪🡪🡪🡪 | | **LMOTI2** | | | |  |
| 14.6 | **Targets**  *O What would need to change for you to want to get PrEP?* | | [Checkbox] | | | 🡪🡪🡪🡪🡪🡪🡪🡪🡪🡪 | | **LMOTI3** | | | |  |
| 14.7 | *Some people are ready to start PrEP. Most people think that it is helpful to have information, no matter how ready they are. So if it is okay with you, I will share some additional information with you:*  O PrEP is a pill for HIV-negative persons to take daily to help them from getting HIV. **[I]**  O PrEP is free.**[I]** O The RHSP X clinic is the nearest PrEP supply location.**[I]** | | [Checkbox] | | | 🡪🡪🡪🡪🡪🡪🡪🡪🡪🡪 | | **L1** | | | |  |
| **M** | **PrEP Module those taking PrEP (Same module as L, but activated according to different inputs)** | |  | | |  | |  | | | |  |
| 14.1 | Please ask the client the following questions using the motivational interviewing skills you have learned. | |  | | | 🡪🡪🡪🡪🡪🡪🡪🡪🡪🡪 | |  | | | |  |
| 14.2 | **Information**  O *Can you tell me what you know about Pre-Exposure Prophylaxis, also known as PrEP?* | | [Checkbox] | | | 🡪🡪🡪🡪🡪🡪🡪🡪🡪🡪 | | **MINFO1** | | | |  |
| 14.3 | **Motivation**  *O What would be some benefits to taking PrEP?*  *O What are your concerns about PrEP?*  *O What have been the not so good things that have happened or may happen by not getting PrEP?* | | [Checkbox] | | | 🡪🡪🡪🡪🡪🡪🡪🡪🡪🡪 | | **MMOTI1** | | | |  |
| 14.4 | **Behavioral Skills**  O *How confident are you that you could get PrEP if you wanted to?*  *O What would make getting the PrEP easy for you if you wanted it?*  *O What would make getting PrEP difficult for you if you wanted it?* | | [Checkbox] | | | 🡪🡪🡪🡪🡪🡪🡪🡪🡪🡪 | | **MBEHA1** | | | |  |
| 14.5 | **Intentions**  *O Given how you feel right now, would you want PrEP within the next month?* | | Yes…………………. 1  No………………….. 0  Not sure……………. 77 | | | If 1, then skip to 3.7  Else, 🡪🡪🡪🡪🡪🡪 | | **MMOTI2** | | | |  |
| 14.6 | **Targets**  *O What would need to change for you to want to get PrEP?* | | [Checkbox] | | | 🡪🡪🡪🡪🡪🡪🡪🡪🡪🡪 | | **MMOTI3** | | | |  |
| 14.7 | *Some people are ready to start PrEP. Most people think that it is helpful to have information, no matter how ready they are. So if it is okay with you, I will share some additional information with you:*  O PrEP is a pill for HIV-negative persons to take daily to help them from getting HIV. **[I]**  O PrEP is free.**[I]** O The RHSP X clinic is the nearest PrEP supply location.**[I]** | | [Checkbox] | | | 🡪🡪🡪🡪🡪🡪🡪🡪🡪🡪 | | **M1** | | | |  |
| **J** | **Mobile Phone** | |  | | |  | |  | | | |  |
| 12.1 | *I will be following up with you over the next several months to continue these sessions. I may call or text you on your mobile phone number to arrange an appointment. Please let me know if your number changes.* | | [Checkbox] | | | 🡪🡪🡪🡪🡪🡪🡪🡪🡪🡪 | | CONTACT | | | |  |
| **K** | **Wrap-Up** | |  | | |  | |  | | | |  |
| 13.1 | O *Do you have any remaining questions or concerns about HIV and staying healthy?*  *O Thank you very much for allowing me to support you.* | | [Checkbox] | | | 🡪🡪🡪🡪🡪🡪🡪🡪🡪🡪 | | WU1  WU2 | | | |  |
| 13.2 | Locate the Log Book form for this participant and enter the following information:  *O* Comments and/or Next Appointment | | [Checkbox] | | | 🡪🡪🡪🡪🡪🡪🡪🡪🡪🡪 | |  | | | |  |
| 13.2 | Counseling complete.  Additional Participants? | | Yes…………………. 1  No………………….. 0 | | | If 1, then back to 1.8  If 0, then continue to 13.3 | |  | | | |  |
| 13.3 | [Close application] | | [Buttons] | | | End of Algorithm. | |  | | | |  |

**Table B. Descriptions of mLAKE trial primary (1°) and secondary (2°) study outcomes.**

|  | **Outcome** | **Description** | **Numerator/Event** | **Denominator** |
| --- | --- | --- | --- | --- |
| **1°** | **HIV care coverage^1^** | Proportion | # linked to HIV care | # HIV-positive |
| **1°** | **ART coverage^1^** | Proportion | # on ART | # HIV-positive |
| **1°** | **MC coverage^1^** | Proportion | # men circumcised | All men age 15-49 |
| **1°** | **HIV Viral Suppression** | Proportion | HIV+ c VL >400 | All HIV+ participants |
| **2°** | **HIV Incidence** | Rate | # new HIV infections | HIV-negative person-years |
| **2°** | **HIV Testing coverage^1^** | Proportion | # ever tested and received HIV results | All study participants |
| **2°** | **Consistent condom use^1^** | Proportion | # using condoms consistently past 12 months | # sexually active with non-stable partner |
| **2°** | **ART treatment failure^2^** | Proportion | *Composite:* Virologic failure OR Mortality OR Lost to follow-up OR Stopped ART | # Initiated on ART |

^1^Self-reported; ^2^In original protocol, ART treatment failure was proposed as a secondary outcome; however, this was not analysed due to difficulty assessing lost to follow-up with programmatic data.

**Table C. Participant characteristics and outcomes at the baseline, mid-study, and end-of-study survey.**

|  | **Baseline Survey (n=2,148)** | | **Mid-study Survey (n=2,533)** | | | | | | **End-study Survey (n=1,903)** | | | | | |  |
| --- | --- | --- | --- | --- | --- | --- | --- | --- | --- | --- | --- | --- | --- | --- | --- |
| **Individual-Level Characteristics** | **Intervention** | **Control** | **Intervention** | | **Control** | | | **p-value** | **Intervention** | | **Control** | | | **p-value** |  |
| **N** | **1,054** | **1,094** | **1,254** | | **1,279** | | |  | **944** | | **959** | | |  |  |
| **Mean (SD) age (years)** | 30.6 (7.71) | 30.1 (7.87) | 30.8 (8.00) | | 30.5 (8.37) | | |  | 31.5 (8.31) | | 31.7 (8.34) | | |  |  |
| **Ageyrs** |  |  |  | |  | | |  |  | |  | | |  |  |
| 15-24 | 253 (24.0) | 296 (27.1) | 315 (25.1) | | 355 (27.8) | | | 0.176 | 224 (23.7) | | 217 (22.6) | | | 0.819 |  |
| 25-34 | 471 (44.7) | 454 (41.5) | 540 (43.1) | | 508 (39.7) | | |  | 370 (39.2) | | 376 (39.2) | | |  |  |
| >=35 | 330 (31.3) | 344 (31.4) | 399 (31.8) | | 416 (32.5) | | |  | 350 (37.1) | | 366 (38.2) | | |  |  |
| **Sex** |  |  |  | |  | | |  |  | |  | | |  |  |
| Female | 535 (50.8) | 511 (46.7) | 569 (45.4) | | 568 (44.4) | | | 0.625 | 450 (47.7) | | 421 (43.9) | | | 0.099 |  |
| Male | 519 (49.2) | 583 (53.3) | 685 (54.6) | | 711 (55.6) | | |  | 494 (52.3) | | 538 (56.1) | | |  |  |
| **Marital status** |  |  |  | |  | | |  |  | |  | | |  |  |
| Married | 664 (63.0) | 613 (56.0) | 748 (59.6) | | 714 (55.8) | | | 0.027 | 591 (62.6) | | 590 (61.5) | | | 0.809 |  |
| Never married | 122 (11.6) | 182 (16.6) | 178 (14.2) | | 230 (18.0) | | |  | 124 (13.1) | | 124 (12.9) | | |  |  |
| Previously married | 268 (25.4) | 299 (27.3) | 328 (26.2) | | 335 (26.2) | | |  | 229 (24.3) | | 245 (25.5) | | |  |  |
| **Educational Status** |  |  |  | |  | | |  |  | |  | | |  |  |
| None | 102 (9.7) | 80 (7.3) | 109 (8.7) | | 77 (6.0) | | | 0.001 | 171 (18.1) | | 134 (14.0) | | | 0.007 |  |
| Primary | 770 (73.1) | 762 (69.7) | 899 (71.7) | | 883 (69.0) | | |  | 601 (63.7) | | 607 (63.3) | | |  |  |
| Secondary/Tertiary | 182 (17.3) | 252 (23.0) | 246 (19.6) | | 319 (25.0) | | |  | 172 (18.2) | | 218 (22.7) | | |  |  |
| **Religion** |  |  |  | |  | | |  |  | |  | | |  |  |
| Christian/Non-Muslim | 888 (84.3) | 894 (81.7) | 1029 (82.1) | | 1047 (81.9) | | | 0.898 | 784 (83.1) | | 783 (81.6) | | | 0.422 |  |
| Muslim | 166 (15.7) | 200 (18.3) | 225 (17.9) | | 232 (18.1) | | |  | 160 (16.9) | | 176 (18.4) | | |  |  |
| **Occupation** |  |  |  | |  | | |  |  | |  | | |  |  |
| Agriculture/Housework | 191 (18.1) | 177 (16.2) | 209 (16.7) | | 214 (16.7) | | | 0.014 | 181 (19.2) | | 163 (17.0) | | | 0.634 |  |
| Bar/Restaurant | 112 (10.6) | 109 (10.0) | 120 (9.6) | | 101 (7.9) | | |  | 78 (8.3) | | 74 (7.7) | | |  |  |
| Fishing | 304 (28.8) | 303 (27.7) | 389 (31.0) | | 379 (29.6) | | |  | 285 (30.2) | | 296 (30.9) | | |  |  |
| Trade/Shopkeeper | 228 (21.6) | 223 (20.4) | 284 (22.6) | | 258 (20.2) | | |  | 208 (22.0) | | 210 (21.9) | | |  |  |
| Other | 219 (20.8) | 282 (25.8) | 252 (20.1) | | 327 (25.6) | | |  | 192 (20.3) | | 216 (22.5) | | |  |  |
| **Male Circumcision** (among men) |  |  |  | |  | | |  |  | |  | | |  |  |
| No | 227 (43.7) | 230 (39.5) | 241 (35.2) | | 237 (33.3) | | | 0.467 | 154 (31.2) | | 184 (34.2) | | | 0.301 |  |
| Yes | 292 (56.3) | 353 (60.5) | 444 (64.8) | | 474 (66.7) | | |  | 340 (68.8) | | 354 (65.8) | | |  |  |
| **HIV Serostatus** |  |  |  | |  | | |  |  | |  | | |  |  |
| Negative | 650 (61.9) | 708 (64.7) | 787 (62.8) | | 832 (65.1) | | | 0.240 | 592 (62.8) | | 625 (65.5) | | | 0.226 |  |
| Positive | 400 (38.1) | 386 (35.3) | 466 (37.2) | | 447 (34.9) | | |  | 350 (37.2) | | 329 (34.5) | | |  |  |
| **HIV Serostatus Awareness** |  |  |  | |  | | |  |  | |  | | |  |  |
| Yes | 1006 (95.4) | 1043 (95.3) | 1184 (94.4) | | 1225 (95.8) | | | 0.223 | 923 (97.8) | | 939 (97.9) | | | 0.730 |  |
| No | 5 (0.5) | 2 (0.2) | 28 (2.3) | | 25 (2.0) | | |  | 7 (0.7) | | 9 (0.9) | | |  |  |
| Has never Tested | 43 (4.1) | 49 (4.5) | 42 (3.3) | | 29 (2.2) | | |  | 14 (1.5) | | 11 (1.2) | | |  |  |
| **In HIV Care** (among all HIV+) |  |  |  | |  | | |  |  | |  | | |  |  |
| No | 91 (22.8) | 94 (24.4) | 73 (15.7) | | 66 (14.8) | | | 0.705 | 23 (6.6) | | 39 (11.9) | | | 0.017 |  |
| Yes | 309 (77.3) | 292 (75.6) | 393 (84.3) | | | 381 (85.2) | |  | 327 (93.4) | | | 290 (88.1) | |  |  |
| **On ART** (among all HIV+) |  |  |  |  | | |  | |  |  | | |  | |  |
| No | 129 (32.3) | 127 (32.9) | 0.846 | 84 (18.0) | | | 85 (19.0) | | 0.700 | 27 (7.7) | | | 41 (12.5) | | 0.039 |
| Yes | 271 (67.8) | 259 (67.1) |  | 382 (82.0) | | | 362 (81.0) | |  | 323 (92.3) | | | 288 (87.5) | |  |
| **HIV viral suppression** (among all HIV+) |  |  |  |  | | |  | |  |  | | |  | |  |
| No | 113 (28.2) | 126 (32.6) | 0.338 | 97 (21.1) | | | 104 (23.6) | | 0.368 | 38 (11.2) | | | 49 (15.4) | | 0.116 |
| Yes | 287 (71.8) | 260 (67.4) |  | 363 (78.9) | | | 337 (76.4) | |  | 300 (88.8) | | | 269 (84.6) | |  |
| **Condom Use with Non-Stable Partner** |  |  |  |  | | |  | |  |  | | |  | |  |
| Always uses condom | 130 (24.9) | 163 (27.7) | 0.349 | 153 (22.9) | | | 187 (27.3) | | 0.014 | 89 (19.7) | | | 96 (20.2) | | 0.859 |
| Inconsistent condom use | 392 (75.1) | 425 (72.3) |  | 515 (77.1) | | | 498 (72.7) | |  | 363 (80.3) | | | 380 (79.8) | |  |
| **# of sexual partners** |  |  |  |  | | |  | |  |  | | |  | |  |
| None | 69 (6.5) | 89 (8.1) | 0.194 | 88 (7.0) | | | 124 (9.7) | | 0.052 | 80 (8.5) | | | 81 (8.4) | | 0.534 |
| 1 | 561 (53.2) | 547 (50.0) |  | 612 (48.8) | | | 605 (47.3) | |  | 511 (54.1) | | | 496 (51.7) | |  |
| 2+ | 424 (40.2) | 458 (41.9) |  | 554 (44.2) | | | 550 (43.0) | |  | 353 (37.4) | | | 382 (39.8) | |  |

**Table D. Self-reported exposure^1^ to Health Scout intervention at mid-study-survey and end-of-study survey by participant characteristics.**

|  | **Mid-Study Survey** | | | | **End-of-Study Survey** | | | |
| --- | --- | --- | --- | --- | --- | --- | --- | --- |
| **Individual-Level Characteristics** | Exposed | Unexposed | Total | P-value | Exposed | Unexposed | Total | P-value |
| **N** | **569** | **1950** | **2519^2^** |  | **580** | **1311** | **1891^3^** |  |
| **Mean (SD) age (years)** | 31.2 (7.67) | 30.6 (8.36) |  |  | 32.7 (7.77) | 31.1 (8.47) |  |  |
| **Ageyrs** |  |  |  |  |  |  |  |  |
| 15-24 | 126 (18.9) | 539 (81.1) | 665 | 0.018 | 99 (22.8) | 336 (77.2) | 435 | 0.001 |
| 25-34 | 258 (24.8) | 782 (75.2) | 1040 |  | 226 (30.4) | 517 (69.6) | 743 |  |
| >=35 | 185 (22.7) | 629 (77.3) | 814 |  | 255 (35.8) | 458 (64.2) | 713 |  |
| **Sex** |  |  |  |  |  |  |  |  |
| Female | 316 (27.9) | 815 (72.1) | 1131 | 0.001 | 301 (34.8) | 563 (65.2) | 864 | 0.001 |
| Male | 253 (18.2) | 1135 (81.8) | 1388 |  | 279 (27.2) | 748 (72.8) | 1027 |  |
| **Marital status** |  |  |  |  |  |  |  |  |
| Married | 358 (24.7) | 1094 (75.3) | 1452 | 0.001 | 380 (32.5) | 791 (67.5) | 1171 | 0.001 |
| Never married | 65 (16.0) | 342 (84.0) | 407 |  | 44 (17.7) | 204 (82.3) | 248 |  |
| Previously married | 146 (22.1) | 514 (77.9) | 660 |  | 156 (33.1) | 316 (66.9) | 472 |  |
| **Educational Status** |  |  |  |  |  |  |  |  |
| None | 45 (24.3) | 140 | 185 | 0.704 | 114 (37.4) | 191 (62.6) | 305 | 0.001 |
| Primary | 403 (22.7) | 1369 | 1772 |  | 374 (31.1) | 828 (68.9) | 1202 |  |
| Secondary/Tertiary | 121 (21.5) | 441 | 562 |  | 92 (24.0) | 292 (76.0) | 384 |  |
| **Religion** |  |  |  |  |  |  |  |  |
| Christian/Non-Muslim | 470 (22.7) | 1597 (77.3) | 2067 | 0.700 | 490 (31.5) | 1067 (68.5) | 1557 | 0.104 |
| Muslim | 99 (21.9) | 353 (78.1) | 452 |  | 90 (26.9) | 244 (73.1) | 334 |  |
| **Occupation** |  |  |  |  |  |  |  |  |
| Agriculture/Housework | 98 (23.2) | 325 (76.8) | 423 | 0.007 | 108 (31.6) | 234 (68.4) | 342 | 0.178 |
| Bar/Restaurant | 65 (29.5) | 155 (70.5) | 220 |  | 56 (37.3) | 94 (62.7) | 150 |  |
| Fishing | 157 (20.6) | 606 (79.4) | 763 |  | 172 (29.8) | 406 (70.2) | 578 |  |
| Trade/Shopkeeper | 137 (25.6) | 399 (74.4) | 536 |  | 134 (32.2) | 282 (67.8) | 416 |  |
| Other | 112 (19.4) | 465 (80.6) | 577 |  | 110 (27.2) | 295 (72.8) | 405 |  |
| **Male Circumcision** (among men) |  |  |  |  |  |  |  |  |
| No | 83 (17.4) | 393 (82.6) | 476 | 0.581 | 88 (26.2) | 248 (73.8) | 336 | 0.624 |
| Yes | 170 (18.6) | 742 (81.4) | 912 |  | 191 (27.6) | 500 (72.4) | 691 |  |
| **HIV Serostatus** |  |  |  |  |  |  |  |  |
| Negative | 297 (18.4) | 1314 (81.6) | 1611 | 0.001 | 292 (24.2) | 916 (75.8) | 1208 | 0.001 |
| Positive | 272 (30.0) | 635 (70.0) | 907 |  | 285 (42.1) | 392 (57.9) | 677 |  |
| **On ART** (among all HIV+) |  |  |  |  |  |  |  |  |
| No | 9 (30.0) | 21 (70.0) | 30 | 0.804 | 3 (50.0) | 3 (50.0) | 6 | 0.812 |
| Yes | 238 (32.2) | 502 (67.8) | 740 |  | 275 (45.2) | 334 (54.8) | 609 |  |
| **Study Arm** |  |  |  |  |  |  |  |  |
| Intervention | 383 (30.8) | 862 (69.2) | 1245 | 0.001 | 358 (38.1) | 581 (61.9) | 939 | 0.001 |
| Control | 186 (14.6) | 1088 (85.4) | 1274 |  | 222 (23.3) | 730 (76.7) | 952 |  |

^1^Exposure defined as any self-report of having been visited and counseled by a Health Scout; ^2^14 participants with missing data are excluded; ^23^12 participants with missing data are excluded.

**Table E. Adjusted intention-to-treat results comparing study arms.^1^**

| **Outcome** | **Mid-Study Survey** | | | | | **End-Study Survey** | | | | |
| --- | --- | --- | --- | --- | --- | --- | --- | --- | --- | --- |
|  | N | Intervention | Control | PRR  (95% CI) | p value | N | Intervention | Control | PRR  (95% CI) | p value |
| **HIV Care Coverage** | 913 | 393/466  (84.3%) | 381/447  (85.2%) | 1.08  (1.03-1.13) | 0.0003 | 679 | 327/350  (93.4%) | 290/329  (88.2%) | 1.09  (1.05-1.13) | <0.001 |
| **ART Coverage** | 913 | 382/466  (82.0%) | 362/447  (81.0%) | 1.09  (1.03-1.15) | 0.0017 | 679 | 323/350  (92.3%) | 288/329  (87.5%) | 1.07  (1.04-1.12) | <0.001 |
| **HIV Viral Suppression** | 901 | 363/460  (78.9%) | 336/441  (76.4%) | 1.06  (1.00-1.13) | 0.06 | 656 | 300/338  (88.8%) | 269/318  (84.6%) | 0.99  (0.95-1.05) | 0.83 |
| **Male Circumcision Coverage** | 1396 | 444/685  (64.8%) | 474/711  (66.7%) | 1.06  (0.97-1.16) | 0.21 | 1032 | 340/494  (68.9%) | 354/538  (65.8%) | 1.05  (0.96-1.15) | 0.28 |

^1^Antiretroviral Therapy (ART) Coverage and HIV Viral Suppression are among all HIV-positive participants; Male Circumcision is among all men regardless of HIV status; PRR=Prevalence Risk Ratio; CI=Confidence Interval. These analyses are adjusted for age and sex only. A model using all covariates differing at baseline with a p<0.10 produced unstable models with unestimable parameters.

**Table F. Unadjusted and adjusted analyses of association of Health Scout exposure with study outcomes.**

|  | **Mid-Study Survey** | | | | | | | **End-of-Study Survey** | | | | | | |
| --- | --- | --- | --- | --- | --- | --- | --- | --- | --- | --- | --- | --- | --- | --- |
| **Outcome** | **N** | **Exposed** | **Unexposed** | **PRR**  **(95% CI)** | **p value** | **aPRR***  **(95% CI)** | **p value** | **N** | **Exposed** | **Unexposed** | **PRR**  **(95% CI)** | **p value** | **aPRR***  **(95% CI)** | **p value** |
| HTS Coverage | 2519 | 562/569  98.8% | 1835/1950  94.1% | 1.05  (1.03-1.06) | <0.001 | -** | -** | 1891 | 579/580999.8% | 1271/1311  96.9% | 1.03  (1.02-1.04) | <0.001 | 1.01  (1.00-1.01) | 0.014 |
| HIV Care Coverage | 907 | 247/272  91.1% | 523/635  82.4% | 1.11  (1.06-1.17) | <0.001 | 1.08  (1.04-1.13) | 0.0003 | 677 | 278/285  97.5% | 337/392  86.0% | 1.13  (1.09-1.18) | <0.001 | 1.09  (1.05-1.13) | <0.001 |
| ART Coverage | 907 | 238/272  90.8% | 502/635  79.1% | 1.12  (1.06-1.19) | <0.001 | 1.09  (1.03-1.15) | 0.0017 | 677 | 275/285  96.4% | 334/392  85.2% | 1.13  (1.08-1.18) | <0.001 | 1.08  (1.04-1.12) | <0.001 |
| HIV Viral Suppression | 895 | 224/270  83.0% | 471/625  75.4% | 1.10  (1.03-1.18) | 0.0056 | 1.06  (1.00-1.13) | 0.059 | 654 | 241/273  88.3% | 326/381  85.6% | 1.03  (0.97-1.09) | 0.34 | 0.99  (0.95-1.05) | 0.83 |
| Male Circumcision Coverage | 1388 | 170/253  67.2% | 742/1135  65.4% | 1.04  (0.95-1.14) | 0.43 | 1.06  (0.97-1.16) | 0.21 | 1027 | 191/279  68.4% | 500/748  66.9% | 1.03  (0.94-1.13) | 0.48 | 1.05  (0.96-1.15) | 0.28 |
| Consistent Condom Use | 1346 | 64/286  22.3% | 276/1060  26.0% | 0.91  (0.73-1.14) | 0.43 | 0.93  (0.74-1.17) | 0.55 | 921 | 45/261  17.2% | 137/660  20.8% | 0.78  (0.59-1.04) | 0.089 | 0.77  (0.58-1.03) | 0.083 |

*Adjusted for sex, age **Unestimable
