# Supplementary material for: Novel community health worker strategy for HIV service engagement in a hyperendemic community in Rakai, Uganda: A pragmatic, cluster-randomized trial
Source: PLoS Med. 2021 Jan 6;18(1):e1003475. doi: 10.1371/journal.pmed.1003475 (PMC7787382; doi:10.1371/journal.pmed.1003475)
Supplement: Attachment — Submitted filename: mLAKE PLOS Medicine Response FINAL.docx [file pmed.1003475.s003.docx]

Larry William Chang, MD, MPH

Associate Professor

Division of Infectious Diseases

Department of Medicine

725 N. Wolfe St., Suite 216

Baltimore, MD 21205

410-929-2001 T

410-502-7029 F

lchang8@jhmi.edu

September 24, 2020

Dear Editors:

We appreciate the opportunity to submit a revised version of our manuscript entitled “**Novel Community Health Worker Strategy for HIV Service Engagement in a Hyperendemic Community in Rakai, Uganda: A pragmatic, cluster-randomized trial”** for your consideration.

We thank the reviewers for their comments which we feel have substantially improved the manuscript. Responses are attached.

Thank you, and please do not hesitate to contact us with any questions or concerns.

Best regards,

Larry William Chang, MD, MPH

Associate Professor of Medicine, International Health, and Epidemology

Johns Hopkins School of Medicine and Bloomberg School of Public Health

Requests from the editors:

Please adapt the data statement to include details for a non-author contact for inquiries.

**Adapted as requested.**

Please quote aggregate demographic details for study participants in the abstract.

**Now quoted.**

Please list the study's primary and secondary endpoints early in the "Methods and findings" subsection of your abstract.

**Now added to abstract.**

In the abstract and elsewhere in the paper, please add p values alongside 95% CI, where available.

**Now added throughout.**

Please add a new final sentence to the "Methods and findings" subsection of your abstract, quoting 2-3 of the study's main limitations.

**New final sentence added as follows:**

***“Study limitations include reliance on self-report for programmatic outcomes and substantial contamination which may have diluted estimates of effect.”***

After the abstract, we will need to ask you to add a new and accessible "author summary" section in non-identical prose. You may find it helpful to consult one or two recent research articles published in PLOS Medicine to get a sense of the preferred style.

**Completed.**

At the start of the "Discussion" section in the main text, please begin the first paragraph with "In this cluster-randomized trial ..." or similar.

**Modified as suggested.**

Please remove the information on funding at the end of the main text. In the event of publication, this information will appear in the article metadata, via entries in the submission form.

**Removed.**

Please adapt the reference call-outs to remove spaces within the square brackets (e.g., "... health services [8,9].").

**Removed.**

In the reference list, please convert italics and boldface to plain text. Where appropriate, 6 author names should be listed, followed by "et al.".

**Re-formatted.**

Please add a completed CONSORT checklist as a supplementary document, referred to early in the methods section (e.g., "See S1_CONSORT_Checklist"). In the checklist, individual items should be referred to by section (e.g., "Methods") and paragraph number rather than by line or page numbers, as the latter generally change in the event of publication.

**Completed and attached.**

Comments from the reviewers:

*** Reviewer #1:

This article, describes the finding from a pragmatic randomized trial on novel Community Health Worker intervention, called "Health Scouts" for HIV Service Engagement defined as (adherence to ART therapy, viral suppression and male circumcision) in a Hyperendemic Community in Rakai,Uganda. One of the strengths of the study is that is uses a Theory based intervention grounded in a Motivational Interviewing Framework. The use of pragmatic, cluster- randomized trial also provides a rigorous evidence on the effectiveness of CHW interventions for people with HIV in improving health outcomes. The use of a mobile application in a hypendemic, low resource setting is also useful to the field. However there are a number of limitations that need to be addressed prior to publication. The article could be strengthened by stating clearly up front the recruitment procedures, clear eligibility for the study population and more in-depth information about the intervention itself. Furthermore there appears to be great loss to follow up on the intervention from baseline to mid study and end study period thus limiting the reliability and validity of the study findings.

Abstract

*	How is self-care defined?

**In the Abstract, we have clarified that “self-reported HIV care” is specifically “self-reported linkage to HIV care”.**

Introduction

*	The introduction begins by describing combination HIV Interventions (CHI) of HIV testing, male circumcision, and antiretroviral therapy. How does the CHW intervention and/or mobile application link to the CHI.

**We have clarified in the Methods and Supplement sections that the CHW intervention and application included counseling modules on several CHI interventions:**

***“A CHI approach was incorporated, with 10 counseling modules targeting both HIV-positive and HIV-negative residents (Table A in S2 Appendix).”***

*	HIV prevalence is 38%--what is the level of viral suppression? Is ART available?

**We have added in the following details on baseline metrics of HIV prevalence, viral suppression, and ART coverage as follows:**

***“We conducted a pragmatic, cluster-randomized trial called mLAKE (mHealth Lakefolk Actively Keeping Engaged) in an HIV hyperendemic fishing community (baseline HIV prevalence ~38%, baseline ART coverage ~67% and HIV viral suppression ~66% among HIV-positive residents) in Rakai, Uganda …”***

Methods

Study design: what is usual care that the Health Scout intervention is compared to?

**We have added additional details on usual care as follows:**

***“Household residents in both study arms had access to standard of care HIV prevention and treatment services which included free HTS, ART, and condoms at a community-based HIV clinic, peer services for patients on ART [24], and access to MC through periodic mobile MC camps***.”

Participant eligibility criteria: If the study is about HIV care coverage and ART therapy—why is the eligibility for this study limited to just people HIV >15 rather than all resident. Or state that this is sub-study of the larger study.

**We have clarified study eligibility as follows:**

***“The RCCS is an open, population-based cohort focused on HIV surveillance among persons 15-49 years of age [25]. Since 2011, the RCCS has surveyed all age-eligible individuals, regardless of HIV status, in this community approximately every 18 months.”***

Participant recruitment: page 7 Health Scouts started visiting "clients". How were clients contacted?

**We have added additional details on recruitment and referred to an additional background paper we failed to previously emphasize as follows:**

***“During each visit, Health Scouts would systematically approach clients at their households and, after receiving oral consent, counsel clients with assistance from the smartphone application.”***

***“The Health Scout intervention and initial implementation have previously been described in detail [17,19].”***

Describe the community mobilization techniques?

**We have added some additional details as follows and referenced the paper which describe community mobilization in further detail:**

***“Community mobilization included informational meetings with community leaders and public drama shows depicting proposed Health Scout activities [19].”***

Did CHWs going randomly from house systematically and inform clients about the program and see if someone wanted to participate.?

**Yes, please see response above on recruitment.**

Phone application Intervention: How many modules?

**10 total modules, which is now noted in the text and provided in the supplement as described above.**

What was the protocol for delivering modules?

**We have edited the “Health Scout Phone Application” section and the supplement to provide more details on delivery, e.g. the entire phone algorithm protocol is now available in the supplement.**

Implementation: How many clusters were each CHW assigned? So Health Scout CHWs were in all 40 clusters? Mobile clusters were only in a few? How many clients in each cluster; how often follow up in 3 month follow-up (weekly?) at least once?

**We have added details as follows:**

***“Health Scouts were each assigned 2 clusters which together contained an average of about 215 eligible clients at baseline.*** ***Health Scouts attempted to visit all clients within their clusters within 3 months of study initiation and follow-up each client within their cluster once every 3 months for ~3 years.”***

**We have also tried to clarify throughout the paper that Health Scouts were in 20 out of the 40 total clusters which were all static.**

Control arm: what was the standard of care?

**Please see response above on the edits to this section.**

Outcomes: If person tests positive are they informed of the results?

**We have clarified that HIV results were returned to study participants as follows:**

***“Following the interview, free HIV testing services with results immediately returned are offered to consenting participants.”***

How is HIV self-care coverage? How is MC coverage measured? How is ART measured?

**Please see above for clarifications that these outcomes were all self-reported.**

Sample size: provide 2-3 sentences about the power calculation and estimated size of each population in the clusters

**We have added these details to the sample size section as follows:**

***“The sample size for this trial was fixed at 40 clusters (20 intervention, 20 control, ~107 eligible participants in each cluster); therefore, we estimated whether the trial had power to detect relevant differences in primary study outcomes, i.e. minimum detectable differences between arms [26]. Detailed power calculation inputs and results were previously described [17]. In summary, the trial was estimated to have the power to detect minimum difference prevalence risk ratios with 80% power of ≥15% differentials in HIV care coverage, ≥20% in ART coverage, ≥41% in viral suppression, and ≥23% in MC coverage.”***

Statistical Analysis: The analysis and results could be strengthened if authors examined the sample that had baseline and follow up data either mid point or end survey.

**We have attempted to clarify in the text that the main analysis for this study was serial cross-sectional, not longitudinal.**

***“A serial cross-sectional analysis approach was chosen a priori as high loss to follow-up was anticipated in this mobile population and our main interest was outcomes across the entire population present at time of survey and not just participants present at baseline.”***

Results-Table

Is there an attrition analysis? According to tables there is significant lost to followup between baseline, mid point and end survey. How many people had completed data ie. at least one baseline and follow up.

**We have added some additional details on attrition in the Figure. However, please note response above regarding primary analysis being serial cross-sectional.**

How did you calculate the response rates for follow time points? Given the samples size at mid year and end study more than 50% of the sample 425 (baseline to end survey) and 413 for controls. AS new participants were recruited at mid year -were they matched to

**Please see comments above. Participants were not matched in this study as this was a cluster-randomized study analyzed cross-sectionally and allowing for in and out migration into static clusters. Response rates were calculated based upon all eligible residents at the time of each survey, not on whether they were present at the prior survey or not.**

Implementation measures: The average of 4 visits over 3 years seems quite low over 3 years. What was the average time in the intervention? Is this just for people with HIV? nearly 25% received only 1 session--why such a big LTF? What was the protocol?

**We were not surprised to see an average of 4 visits nor 25% receiving only 1 session due to the high mobility of study participants. We agree that average time in the intervention is of interest and were planned for about 30-45 minutes in the original protocol. We hope to present those process results in detail in subsequent publications focused on implementation-specific processes and outcome as there is insufficient space to properly address all of these issues in this publication focused on primary outcomes. Please also see response above noting this intervention was for both HIV-negative and HIV-positive participants.**

Only 38% of the intervention group received the Health Scout intervention with the mobile app. This seems low for an intervention design.

**The issue of reach is complicated by the likely contamination.** **That is, if there had been no contamination, and we had 100% reach in the intervention clusters and 0% in the control clusters, the overall reach in the entire community would be maxed at 50%. This study found 30% reach (i.e. 60% of maximum possible reach) across the entire community which is good for a community-based intervention. We have added additional comments on reach to the Discussion section as follows:**

***“Based on self-report, intervention reach in this study was moderate with only 38% of residents reporting receiving counseling by the Health Scouts in the intervention arm by study end (of note, if there had been no contamination and 100% reach in the intervention clusters, the overall reach in all 40 clusters would be 50% maximum, what was found in this study considering contamination was 30% overall community reach).”***

For those who are not HIV infected what were their outcomes of interest? It is not clear why they are included in this study.

**For HIV-negative individuals, male circumcision coverage was the main outcome of interest. Sexual behavior change, specifically condom use, was a secondary outcome as well as history of HIV testing and HIV incidence. HIV-negative individuals were included in the study because they have HIV prevention service needs and also to prevent stigmatization and disclosure issues which may arise from only counseling HIV-positive individuals in a community-based, household-based intervention.**

Discussion

Is the intervention sustainable? It seems there was high turnover among Health SCOUTS from 10 to approximately 5?

**We agree that issues of sustainability are critical. We note that we began with 10 Health Scouts and ended up with 8 which was sufficient for the workload. Given the high mobility of this population, we were not surprised by some Health Scouts leaving or being able to readily recruit additional Health Scouts. We hope to further address sustainability issues in subsequent mixed methods publications which will provide further analyses which we were not able to fit in this publication.**

Limitations: The authors duly note that contamination could be reasons for null effects between intervention and control groups. Was supervision provided to Health Scouts to try to limit contamination.

**Yes, Health Scouts were supervised and instructed on avoiding contamination repeatedly. This was described in detail in an earlier publication which we have now better referenced throughout. Text was also added as follows:**

***“10 Health Scouts were initially elected and recruited from the community and underwent residential- and field-based training on application and smartphone use, confidentiality, disclosure, HIV-related knowledge, cluster boundaries, and avoiding contamination.”***

What further recommendation for future study designs and would the authors recommended? Step Wedge?

**We have added the following to the Discussion:**

***“Future studies may consider having entire communities be the unit of randomization to decrease contamination risks.”***

*** Reviewer #2:

PMEDICINE-D-20-03569R1

This pragmatic cluster RCT examines the effectiveness of a community-based motivational interviewing m-health intervention embedded within the Rakai cohort. The study design and careful plan are strengths. However, there are some loose ends that would be worth clarifying further. In particular, more discussion of the pilot data, contamination, LTFU, and intervention would enhance the value of this study.

MAJOR COMMENTS

(1)	Pilot data. Given that there was no difference between the two arms, it would be helpful to know about the pilot data that informed the sample size calculation and study design. However, I could not find published pilot data demonstrating the effectiveness (beyond feasibility) of this specific intervention in the protocol manuscript or this submitted paper. Could this data be highlighted?

**The data informing this intervention were based on several prior studies of community health workers and mobile health tools which our group conducted in Uganda. We have added references and explanation as follows:**

***“…we estimated whether the trial had power to detect relevant and realistic differences in primary study outcomes, i.e. minimum detectable differences between arms, based on our prior experience with community health worker interventions and mobile health tools in Uganda [24,26-28].”***

(2)	Contamination. The extent of contamination in Figure 1 seems rather modest. While I agree this would bias towards the null, the sample size calculations accounted for 10-50% contamination. A more thorough discussion of contamination and mention of this in the abstract would be useful for the reader.

**We agree that contamination suggested by Figure 1, i.e. participants moving from intervention to control arms and vice versa, is relatively modest. However, contamination by self-reported receipt of the intervention was substantial, i.e. 23% of control arm participants reported receiving the intervention compared to 38% in the intervention arm. We have attempted to emphasize and explain these findings as follows in the abstract:**

***“At end-study, self-reported receipt of the Health Scouts intervention was 38% in the intervention arm and 23% in the control arm, suggesting moderate intervention uptake in the intervention arm and substantial contamination in the control arm.”***

**and discussion:**

***“Participant flow (Figure 1) from intervention to control arms and vice versa appeared modest; however these data only captured residence at time of survey and participants may move residence more than once during inter-survey intervals, e.g. many participants rely on short-term rentals or transient sleeping under boats.”***

(3)	Rakai cohort LTFU and migration. The study was nested within the Rakai open cohort, making this one of the most extensively researched populations in the world. Given this context, it is surprising that migration and LTFU were such unanticipated problems. Did something change with the cohort over time or is there some other explanation for these differences?

**We were not overly surprised by the high levels of migration and LTFU in these fishing communities (which were added in 2011 to the much less mobile, general population Rakai cohort). Indeed, that is why we choose serial cross-sectional analyses as our primary analyses of interest as we knew following only those present from baseline to end-study would be underpowered. We have clarified this as follows:**

***“A serial cross-sectional analysis approach was chosen a priori as high loss to follow-up was anticipated in this mobile population and our main interest was outcomes across the entire population present at time of survey and not just participants present at baseline.”***

(4)	Health Scouts. More details on these individuals either in the text or a supplement would be useful. Based on looking at the protocol, they had no experience with motivational interviewing and were tasked with implementing a substantial public health task with minimal mentorship. How was "basic competency with assigned tasks" evaluated in the field? What support was provided and how were they compensated?

**We should have better referenced a companion paper (Long et al, 2017) to the protocol which was previously published and describes training, skill assessment, and supervision in much more detail. We have added in a few details in this paper throughout and emphasized the reference. For example:**

***“Motivational interviewing skills, a communication strategy that emphasizes a non-judgmental and non-confrontational approach to behavior change, were integrated into training with core competencies evaluated [19,22,23].”***

(5)	M-health data. Was the back-end data from app use also used to see levels of use? This implementation data might be able to what extent scouts were implementing the standard intervention.

**We agree this would be interesting to look at and it is possible. However, the mobile phone-based data is complicated to both analyze and explain, and we believe would be better served through its own dedicated publication which we are actively working on.**

MINOR COMMENTS

(1)	Abstract, results section. Contamination. Given that contamination was an important part of this study, including some basic description of the scope of contamination in the abstract would be important.

**We agree and have added the following:**

***“At end-study, self-reported receipt of the Health Scouts intervention was 38% in the intervention arm and 23% in the control arm, suggesting moderate intervention uptake in the intervention arm and substantial contamination in the control arm.”***

(2)	Abstract, results section, sentence starting with "At end-study, self-reported.." I believe that the counselling was the intervention itself, correct? Would consider revising this sentence to focus on exposure to the intervention in the two study arms.

**We agree. Thank you for this suggestion, please see above response.**

(3)	Secondary outcomes. It would also be useful to see the pre-specified secondary outcomes of interest (condomless sex, viral load, testing coverage, etc).

**We have included secondary outcomes in the revised manuscript and supplement.**

(4)	Per protocol analysis. The protocol published in Trials does not specify a per-protocol analysis. Was this added later? It's a nice addition to the paper, but would be appropriate to note that this was not pre-specified (if this was the case).

**In response to both this comment and the one provided by the statistical reviewer, we have now included a per protocol analysis which we had always intended to do but failed to state in the original protocol paper. Please see supplement for results of this analysis. Relevant text in the main paper includes the following:**

***“Finally, an unadjusted and adjusted (for age and sex) as-treated analysis found that self-reported exposure to the Health Scout intervention was associated with increased HTS, HIV Care, and ART coverage at both mid-study and end-study and increased HIV viral suppression at mid-study compared to unexposed participants.”***

(5)	Figure 2S. Its great to have some screen shots, but would be good to have a larger high-resolution version of this content.

**We have enlarged the screen shots in the supplement.**

*** Reviewer #3:

Alex McConnachie, Statistical Review

Chang et al present the results of a cluster randomised trial of a community health worker intervention to improve HIV service engagement in a hyperendemic setting in Uganda. This review considers the use of statistics in the paper.

Overall, I found the paper to be interesting, and well written. The study background, methods, and results, are described clearly.

The sample size justification is reasonable; studies like this often have a fixed sample size, and the question is whether the study has power to detect a reasonable effect. There is a reference to another paper with details of the sample size calculations, which is fine, but I think in the current paper it would be good to state what minimum effect size the study was powered to detect.

**We have added these details as follows:**

***“The sample size for this trial was fixed at 40 clusters (20 intervention, 20 control, ~107 eligible participants in each cluster); therefore, we estimated whether the trial had power to detect relevant and realistic differences in primary study outcomes, i.e. minimum detectable differences between arms, based on our prior experience with CHW interventions and mobile health tools in Uganda [24,26-28]. Detailed power calculation inputs and results were previously described [17]. In summary, the trial was estimated to have the power to detect minimum difference prevalence risk ratios with 80% power of ≥15% differentials in HIV care coverage, ≥20% in ART coverage, ≥41% in viral suppression, and ≥23% in MC coverage.”***

The statistical analysis methods are acceptable, as far as they go. One of the most striking results is the level of contamination between the study arms, with almost a quarter of the control arm reportedly receiving the intervention. Whilst an ITT analysis is perfectly reasonable, I feel the authors should seek to estimate what the true impact of the intervention might be. For example, this could involve a CACE analysis to estimate the effect of the intervention amongst those who received it. This, coupled with the estimated reach of the intervention, could give an estimate of the impact of the intervention over a population. Currently, we have estimates of differences in outcomes between two groups with different levels of access to the intervention, which provide evidence of intervention effects, but do not give good estimates of the magnitude of those effects. What matters, I think, are the differences in outcomes between a population with access to the intervention and the same population without access to the intervention.

**We have added a new per protocol analysis to the supplement and reference it in the main text as follows:**

***“Finally, an as-treated analysis was conducted with exposure defined as self-reported receipt of any Health Scout counseling (exposure data was also collected through the RCCS survey).”***

***“Finally, an unadjusted and adjusted (for age and sex) as treated analysis found that self-reported exposure to the Health Scout intervention was associated with increased HTS, HIV Care, and ART coverage at both mid-study and end-study and increased HIV viral suppression at mid-study compared to unexposed participants.”***

*** Reviewer #4:

This paper reports the primary outcomes of a cluster-randomized trial evaluating the effect of a community health workers intervention (called "Health Scouts") on engagement into HIV care and prevention measured at the population level. The trial was embedded into a population cohort used to measure the different outcomes at baseline, mid-point and end line. Implementing and evaluating complex combination interventions are crucial to achieving epidemic control. The results reported in this paper are entirely relevant in this context.

Overall, the Health Scouts intervention could be more described in the paper, the authors referring to a protocol paper published in Trials (<https://doi.org/10.1186/s13063-017-2243-6>).

**We should have better referenced a companion paper (Long et al, 2017) to the protocol which was previously published and describes training, skill assessment, and supervision in much more detail. We have added in a few details in this paper throughout and emphasized the reference. For example:**

***“Motivational interviewing skills, a communication strategy that emphasizes a non-judgmental and non-confrontational approach to behavior change, were integrated into training with core competencies evaluated [19,22,23].”***

In particular, it is unclear what are the counselling messages delivered by the scouts. The paper is focused on the 4 primary outcomes of the trial, 3 of them concerning only HIV-positive individuals (HIV care coverage, ART coverage and viral suppression) and one among men (male circumcision). When just reading the paper, it seems strange that all individuals were eligible for the scout intervention as it was unclear that would be the counselling for negative women.

**We have now added to the supplement the entire counseling algorithm so that readers may see all the counselling messages used by the Health Scouts. We have also attempted to clarify in the text that counseling included specific messaging for HIV-negative men and women, e.g. regarding PrEP. Finally, secondary outcomes have been added to the main text and supplement.**

In the protocol paper, it appeared that there are 9 counselling modules: 1 HIV serostatus unknown or no recent HIV test, male or not pregnant female; 2 HIV serostatus unknown or no recent HIV test, pregnant female; 3 Male, not circumcised; 4 HIV+, not in care; 5 HIV+, in care, not on antiretroviral therapy (ART); 6 HIV+, on ART; 7 HIV+, pregnant; 8 Male having condomless sex; 9 Female having condomless sex. However, it is not described whare are the recommendations for each group. For example, are uncircumcised HIV-positive men invited to get circumcised?

**The supplement now includes the full counseling protocols and messaging for each module. There was also an additional PrEP module which was added after the initial protocol.**

According to the protocol paper, there are 4 defined secondary outcomes (HIV testing, HIV incidence, HIV treatment failure and condom use). What are the reasons for not reporting these 4 outcomes in the same paper as the 4 primary outcomes? It would provide a more global view of this global and multi-component intervention.

**We originally did not report secondary outcomes out of a concern for space limits. In this revision, we have added secondary outcomes to the supplement. Relevant text is as follows:**

***“Analysis of secondary outcomes did not find any significant differences in HTS coverage, condom use, or HIV incidence at mid-study or end-study.”***

The paper is relatively silent about what was done by the health scouts, just mentioning 11,221 counselling sessions among 2,532 unique residents. Do the authors have any information/statistic about the content of the sessions? Was data collected in the app about the profile of the beneficiaries?

**We agree and have emphasized the initial implementation paper (Long et al, 2017) which provides further details, as well as provided the detailed counseling algorithm. We did collect data on the application; however, the mobile phone-based data is complicated to both analyze and explain, and we believe would be better served through its own dedicated publication which we are actively working on.**

Regarding the 4 primary outcomes, they should be more formally defined, with a precise definition of the numerators and the denominators.

**We have now provided specific definitions in the supplement of all study outcomes.**

It appears in the Results that 3 surveys have been conducted at baseline, mid-point and endline. However, the baseline survey is not mentioned in the methods. Was it also conducted as part of RCCS?

**We have clarified these points in the methods section as follows:**

***“The study was nested within the Rakai Community Cohort Study (RCCS) which was used to assess baseline characteristics and all study outcomes through three community-wide, cross-sectional surveys, one at baseline (~4 months prior to intervention start), one at the study mid-point (~15 months after intervention start) and one at the study end-point (~39 months of total follow-up).”***

Regarding the analysis of the primary outcomes, it seems that only within-cluster correlations were taken into account, but that the models were not adjusted on sociodemographic characteristics. In the protocol paper, it is written: "If a baseline outcome or demographic variable are significantly different between arms with p-value ≤ 0.1, adjusted regression analysis on the outcome variables will be conducted adjusting for baseline covariates." According to table 1S, at baseline, the two arms differ statistically (p < 0.1) in terms of sex, marital status and educational status. Therefore, could the authors justify the choice of non-adjusting on these variables?

**We did conduct an adjusted analysis but as the outcomes did not differ significantly from the unadjusted analysis we decided to present the later. However, we have now noted this adjusted analysis in the main text and included a Table of the adjusted analysis in the supplement.**

Considering the fact that only a small proportion (38%) of participants self-reported health scouts counselling at endline in the intervention arm and that 23% of participants reported health scouts counselling in the control arm, did the authors consider the possibility of a comparison of the different outcomes between those reporting counselling vs the others, as a complementary analysis to the intent-to-treat analysis?

**We have now included a new as-treated analysis. Please see responses above and revised supplement.**

The authors may consider some rewording of the two paragraphs presenting the intention-to-treat analyses as it is confusing in its current form. The two paragraphs start with the number of HIV-positive participants, suggesting that this subgroup was the denominator of the different outcomes. But this is true only for 3 outcomes (HIV care coverage, ART coverage and viral load). Clearly, male circumcision coverage used a different denominator, not mentioned in the text.

**We thank the reviewer and have adjusted the paragraph as follows:**

***“At the mid-study survey, among 1396 men, male circumcision coverage did not differ by study arms.”***

***“HIV viral suppression among HIV-positive participants and male circumcision among all men were numerically higher in the intervention compared to the control arm but the difference was not statistically significant.”***

Also, could the authors clarify if male circumcision coverage was computed among all men or only among those HIV-negative?

**We have clarified that male circumcision coverage was computed among all men.**

In the discussion, there is the following statement: "The intervention arm in this study was able to achieve these benchmarks" (i.e. 95-95-95 Unaids' targets). However, data to justify this statement are not presented in the paper. Besides, achieving these targets at endline without knowing what was the situation at baseline nor what was achieved in the control arm, doesn't mean anything about the contribution of Heath Scouts in this achievement.

**We have removed this from the Discussion.**
